# The Role of Citizen Science in Promoting Health Equity

**DOI:** 10.1146/annurev-publhealth-090419-102856

**Published:** 2021-11-01

**Authors:** Lisa G. Rosas, Patricia Rodriguez Espinosa, Felipe Montes Jimenez, Abby C. King

**Affiliations:** 1 Department of Epidemiology and Population Health and Department of Medicine, Stanford University School of Medicine, Stanford, California, USA; 2 School of Engineering, Universidad de los Andes, Bogotá, Colombia

**Keywords:** citizen science, health equity, participatory research, environmental health, community, physical activity, healthy eating

## Abstract

While there are many definitions of citizen science, the term usually refers to the participation of the general public in the scientific process in collaboration with professional scientists. Citizen scientists have been engaged to promote health equity, especially in the areas of environmental contaminant exposures, physical activity, and healthy eating. Citizen scientists commonly come from communities experiencing health inequities and have collected data using a range of strategies and technologies, such as air sensors, water quality kits, and mobile applications. On the basis of our review, and to advance the field of citizen science to address health equity, we recommend (*a*) expanding the focus on topics important for health equity, (*b*) increasing the diversity of people serving as citizen scientists, (*c*) increasing the integration of citizen scientists in additional research phases, (*d*) continuing to leverage emerging technologies that enable citizen scientists to collect data relevant for health equity, and (*e*) strengthening the rigor of methods to evaluate impacts on health equity.

## INTRODUCTION

There is an urgent need for new approaches to effectively mitigate widespread and persistent health inequities both in the USA and globally, with the ultimate goal of achieving health equity. Although progress has been made in improving overall health outcomes in the USA, inequities in leading causes of death such as cardiovascular disease, cancer, and diabetes have persisted and, in some cases, increased over time (31). Health inequities are the result of a complex interplay of factors at the individual, family, community, and policy levels. Individual-level interventions, such as those that promote healthy lifestyles for chronic disease prevention and management, are critical and have shown promise in improving health outcomes (16, 38). However, in the absence of changes to the physical and social environments, these improvements can be challenging to achieve and almost impossible to sustain. Theoretical frameworks, such as social cognitive theory and the socio-ecological model, acknowledge the importance of modifying the physical and social environments as critical components of improving population health and achieving health equity (2, 49).

Developing and testing such multilevel strategies are challenging within existing biomedical research paradigms. Existing paradigms often result in deficit-based interventions and culturally inappropriate practices and have struggled to achieve policy or system changes (25). Likewise, limited participation of community members affected by health inequities can result in research findings that are not relevant or applicable for mitigating health inequities. Community members and other stakeholders (e.g., policy makers, teachers, health care providers) bring their lived experience of health inequities and are embedded in the physical and social environments that are important to change. Finally, existing biomedical research approaches often fail to account for the immense diversity in the social, cultural, demographic, and geographic factors that are particularly important for achieving health equity.

Participatory research models offer an alternative to existing biomedical research models. Citizen science is an emerging model of participatory research in the health field that is increasingly employed to address health equity. While there are many definitions of citizen science, the term usually refers to the participation of the general public in the scientific process in collaboration with professional scientists (20, 46). Citizen science dates back to the American Founding Fathers, with the use of the term citizen referring to inhabitants of a particular locale without regard to legal status. In modern times, citizen science is also referred to as community science. Because much of the extant literature has used the term citizen science, we use that term throughout this review, keeping in mind the broader definition of citizen, above.

Reflecting the range of public involvement in the scientific process, several taxonomies of citizen science have been put forward. Rowbotham et al. (46) have proposed the following three levels of citizen science: (*a*) contributory, (*b*) collaborative, and (*c*) co-created. Contributory citizen science involves citizen scientists in data collection only. Collaborative citizen science extends community member involvement to data analysis and interpretation. Co-created citizen science further extends involvement to defining the problem and translating research findings into public health impact. English et al. (20) have proposed an analogous taxonomy, also with three levels: (*a*) crowdsourcing, (*b*) limited participatory research, and (*c*) extreme citizen science. Crowdsourcing, similar to contributory citizen science, refers to active or passive participation in data collection, such as contributing data through self-monitoring or through personal sensors or other forms of technology. Limited participatory research, similar to collaborative citizen science, involves the community in problem definition and data collection. Extreme citizen science, similar to co-creation, also involves the community in analysis and interpretation, study dissemination, and public health action. A third taxonomy, proposed by King et al. (37), similarly defines three levels of citizen science as (*a*) for the people, (*b*) with the people, and (*c*) by the people. Citizen science “for the people” is similar to contributory citizen science and focuses on individual contributions of biological samples or other personal health information. Citizen science “with the people,” similar to collaborative participatory research and popular in the natural and ecological sciences, includes opportunities for the public to actively participate in a standardized data collection process (e.g., local bird counts), with the data pushed to scientists who then analyze and interpret the data. Citizen science “by the people” is analogous to co-created or extreme citizen science and aims to involve community members in all phases of the research process, including involving local decision makers in applying the data to inform and activate community action (37).

Citizen science approaches with a high level of involvement of community members (e.g., “by the people”) are aligned with a community-based participatory research (CBPR) orientation (41). The alignment centers on a shared goal of integrating community perspectives throughout the research process and a focus on health equity. As such, citizen science with a high level of involvement of community members can be conducted within a CBPR partnership. It is useful to note that numerous approaches fit within a CBPR orientation, while citizen science refers to a specific methodology. Citizen science approaches historically center on community members employing systematic and/or rigorous forms of data collection. As such, citizen science offers a model for engaging directly with individual community members outside of the formal community–university partnerships that are often essential for CBPR. This overcomes the need for formal infrastructure in communities (i.e., community organizations that are willing and able to play a primary role) and enhances research participation when there are insufficient community-based organizations that represent a certain group or health topic, or during emerging health inequities such as those related to the coronavirus 2019 (COVID-19) pandemic. Additionally, many forms of citizen science are particularly concerned with physical and social environmental contexts and their impacts on human and/or planetary health and well-being.

The power of citizen science with greater levels of involvement lies in the opportunity for community members to identify, systematically collect, analyze, and utilize data that are meaningful and relevant to them. This is highly relevant for promoting health equity, as the influence of social and physical environments on health inequities is often only noticed by those for whom it is unfair and unjust and may be less well understood by those in control of a community’s decision-making levers or channels. This review focuses on citizen science that involves community members in more than solely collecting or contributing data. The overall goals of this review are to summarize existing efforts using citizen science to address health equity and to provide recommendations to advance the field.

## METHODS

To synthesize the literature, we conducted a keyword search of the extant published literature using PubMed, PsycINFO, and CINAHL. Keywords included the following: (“citizen science” OR “community science” OR “participatory science” OR “citizen scientist”) AND (“health promotion” OR “health behavior” OR “public health” OR “social environment” OR “eating” OR “exercise” OR “health” OR “nutrition” OR “social connection”). As shown by the keywords, citizen science/scientist and similar terms (e.g., community science) were included. Whenever possible, keywords were used as medical subject heading (MeSH) terms (e.g., citizen science, health promotion, health behavior, public health, eating, exercise) or synonyms to capture similar terms and concepts. For example, physical activity is an entry term already included under “exercise” as a MeSH term. We imposed no time period limitations in order to capture as many relevant papers as possible. We identified a total of 425 articles and screened the abstracts using predefined inclusion and exclusion criteria. To be included, a study had to focus on human health and involve citizen scientists in more than solely data collection. Review articles, commentaries, and conceptual pieces were excluded. We selected 107 of these 425 articles for full-text screening and analysis, applying the above initial inclusion and exclusion criteria as well as assessing whether the article addressed health equity. To qualify for addressing health equity, each study had to address a health determinant or outcome in a community that experienced a disproportionate burden of disease that was considered unfair or unjust in accordance with definitions of health equity (3). Of the 107 articles selected for full-text review, 22 were identified. We then reviewed the reference sections of these 22 articles and identified 42 additional articles for full-text review. We applied the same inclusion and exclusion criteria to these 42 additional articles and identified 5 articles to be included, for a total of 27 articles. We examined these 27 articles and summarized the following: (*a*) research frameworks guiding the citizen science process, (*b*) common research areas, (*c*) tools used to facilitate data collection, (*d*) characteristics of citizen scientists engaged in research, (*e*) inclusion of citizen scientists in various phases of research, and (*f*) methods to evaluate the process and outcomes of engaging citizen scientists.

## RESULTS

Of the 149 articles that qualified for full-text screening, 27 were included in our final selection (Table 1). Most of the excluded articles (31%) were conceptual or review articles or did not report sufficient details of an actual citizen science project (e.g., peripheral mention of a citizen science effort, or preparation for a future project). Another 19% were excluded for involving citizen scientists only in data collection, with no other involvement in the research process (i.e., they did not reflect the tenets of co-created, extreme, or “by the people” citizen science); 12% were not related to health equity; 9% were unrelated to citizen or participatory science; and 8% were not related to human health. Two-thirds (67%) of the included papers were published in 2018 or later.

### Frameworks

Several research frameworks, models, and methods were mentioned across the 27 studies included in this review (Table 1). The *Our Voice* citizen science method was commonly used in the research areas of physical activity and healthy eating. *Our Voice* is a technology-enabled, community-engaged global research initiative with the goal of empowering residents of diverse communities to both document and improve features of their physical and social environments affecting various aspects of healthy living (37). Citizen scientists then learn to interpret and prioritize their data and use their findings to engage with decision makers and advocate for improvements at the community level. In addition to the *Our Voice* method, other citizen science studies used participatory research orientations such as CBPR and Youth Participatory Action Research. One study used the Community Health Engagement Survey Solutions (CHESS) framework, which is a systematic approach for engaging citizen scientists in data collection, priority setting, and policy change (27).

### Common Research Areas

The most common research areas in which citizen science was applied were environmental contaminant exposures, physical activity, and healthy eating. Citizen science was also used to address physical and social environments in areas such as implementation of age-friendly environments and the promotion of well-being and reduced stress.

#### Environmental contaminant exposures.

The most common research area (33%) focused on addressing environmental contaminant exposures. Several of the studies engaged citizen scientists in testing household and neighborhood samples for environmental contaminants (22, 32, 43). For example, Ramirez-Andreotta et al. (43) partnered with citizen scientists in a community in Arizona adjacent to a mining operation and a Superfund site to address exposure to arsenic in soil and drinking water within the context of a CBPR partnership. Citizen scientists were trained to collect soil samples in their home gardens and were subsequently involved in advocating for policy changes related to arsenic in their drinking water (43). In a project assessing exposure to secondhand tobacco smoke, citizen scientists in Kentucky partnered with researchers to collect data on indoor air quality using wearable AirBeam monitors and then used data to advocate for smoke-free policies (22).

Three of the studies engaged youth as citizen scientists to address environmental contaminant exposures (26, 28, 57). For example, Wong et al. (57) described a process in which youth were involved in establishing a community network of low-cost air sensors and trained to conduct and translate research into policy changes for their community in the Imperial Valley of California. Similarly, Hahn et al. (26) engaged youth and their teachers as citizen scientists to use home radon testing kits in their communities. The involvement of citizen scientists resulted in a high uptake of radon testing and also increased the youth’s scientific literacy.

Two studies addressed disasters or disaster planning. Sullivan et al. (50) described the process whereby fishermen were trained as citizen scientists following the *Deepwater Horizon* oil disaster off the Gulf Coast to measure exposure to petrogenic polycyclic aromatic hydrocarbons, understand their toxicity, and communicate risks. The authors commented that the involvement of fishermen promoted the rigor of the methods and improved overall environmental health literacy in the community. Newman et al. (39) worked with citizen scientists in communities adjacent to concentrated areas of industrial land use in Texas that are exposed to elevated levels of pollutants during flood disasters. Citizen scientists were involved in data collection as well as in land use changes to protect community members from exposures in future flood disasters (39).

#### Physical activity.

The next most common research area (30%) centered around the promotion of active living, especially in neighborhoods with unfavorable environments for physical activity. Studies engaged children and adults from diverse backgrounds in the USA (44, 56) and globally (40, 45, 58). Several studies assessed environmental features related to physical activity, such as walkability, presence of trash, safety, maintenance of sidewalks and other walking paths, and access to recreational or exercise facilities, including parks (45, 51, 55). For instance, Rodriguez and colleagues (44) engaged Latinx school-age children and parents in assessing the built environment for barriers related to Safe Routes to School programs and demonstrated increases in the number of children and families utilizing alternative and healthful modes of commuting (e.g., biking, walking). Global efforts included engagement of citizen scientists in three countries—the USA, Colombia, and Chile—in assessing the impacts of publicly available physical activity initiatives such as pop-up parks and street closures to promote active living (58). Other studies assessed the impacts of similar initiatives featuring community-based physical activity promotion programs and their potential uptake and scaling (47). Taken together, these studies show the feasibility of employing citizen science methods across countries and populations to evaluate opportunities for increased active living and advocacy for enhanced infrastructure in communities with histories of disinvestment (40, 45, 51, 58). Additionally, studies reported engagement efforts that included community residents as citizen scientists as well as other key stakeholders (e.g., policy makers, teachers, health care providers). For example, Tuckett and colleagues (51) engaged with city council members in Brisbane, Australia, as a strategy to disseminate information to stakeholders with jurisdiction and resources to make repairs and improvements in areas of the built environment supporting physical activity. Winter and colleagues (56) engaged residents, business owners, and elected officials and other decision makers in a comprehensive evaluation of city-based pop-up parks in Los Altos, California.

#### Healthy eating and nutrition.

The studies employing citizen science to address healthy eating (22%) tended to focus on nutrition environments. Two studies focusing specifically on food access in underserved communities used the *Our Voice* method (14, 48). For example, Sheats et al.(48) used the *Our Voice* method to engage ethnically diverse lower-income older adults in the San Francisco Bay Area to collect data about aspects of their local neighborhoods and communities that facilitated or hindered healthy eating. The citizen scientists identified access to affordable healthy foods and transportation as the main barriers. Kim et al. (33) engaged youth members of the Karuk Tribe in Northwest California in a training on research methods. The youth subsequently designed and implemented a community health and food assessment survey, which demonstrated that access to healthy food was a significant issue in their community. Akom et al. (1) engaged youth in East Oakland, California, using several strategies, including a mobile application called Streetwyze. The youth identified unhealthy food choices in retail outlets and food insecurity as barriers to healthy eating in their neighborhood, and used those data to advocate for programs such as a school-based farmers market and a central food commissary that includes kitchen space, a healthy food education center, and an urban farm (1). Hancock et al. (27) utilized the CHESS framework with more than 5,000 citizen scientists in Scotland and England. The process led to several strategies to improve healthy eating, including cooking lessons, gardening, and healthy lunches for schoolchildren.

#### Other health promotion areas targeting social and physical environments.

Some examples of citizen science applications were found in other health promotion domains. Chrisinger & King (13) worked with diverse adults and employed the Empatica E4 wrist-worn sensor with the *Our Voice* method to collect geocoded pictures and narratives to identify key elements of the built environment implicated in stress-related responses, a mechanism posited to underlie the development and maintenance of health inequities. Chesser and colleagues (10, 11) reported the use of citizen science to promote age-friendly environments (e.g., accessibility, signage, transportation, social isolation) in a university environment in Canada by engaging diverse older adults as citizen scientists, as well as other university stakeholders for data collection, and created a formal working group to advocate for institutional change. Garcia and colleagues (23) engaged with diverse homeless youth living in Los Angeles, California, to develop a survey around neighborhood, education, and other issues prioritized by the youth. Youth played a leading role in data collection and dissemination and in advocacy efforts, which included engagement with local decision makers (e.g., the police department, a state senator, the mayor’s office, and city council members).

### Tools for Data Collection

Citizen scientists have used various strategies and technologies for data collection (for more details on commercially available tools used in the studies included in this review, see the Supplemental Material). For studies on environmental contaminants, citizen scientists used various environmental exposure assessment tools, such as air sensors, home sampling kits, and water quality test kits. Notably, a study by Jiao et al. (32) combined exposure assessments with additional layers of publicly available data on health and environmental outcomes. In several of the studies addressing physical activity and healthy eating, citizen scientists used mobile applications and tools to collect data. These mobile applications are able to capture both community assets and barriers to healthy living. Studies that used the *Our Voice* method employed the Stanford Healthy Neighborhood Discovery Tool (Discovery Tool) (7) to take geocoded photographs and record/write audio narratives of features of local physical and social environments that affect health. The Discovery Tool is a mobile application available in multiple languages (e.g., English, Spanish, Chinese) that has been tested in countries spanning six continents, with users spanning a wide range of ages, levels of literacy, and technology comfort (35, 36). Using a different citizen science method, youth citizen scientists in East Oakland, California, used a commercially available mobile application called Streetwyze that enables citizen scientists to collect time-stamped and geocoded information that affects health. Hancock et al. (27) described the CHESS framework, which uses a mobile tool on tablets. Citizen scientists use the tool to collect quantitative and qualitative data about the community and its assets. The tool enables citizen scientists to record details of the food environment, such as fruit and vegetable selection and presence of alcohol advertisements.

### Characteristics of Citizen Scientists

The number of citizen scientists involved in each study ranged from 8 to approximately 5,000, with most studies including 10–30 community members. In some cases, the number of citizen scientists was not reported (28, 39). Given the focus on health equity, citizen scientists tended to be recruited from low-income, racial/ethnic minority, and other underresourced communities. In the case of studies on environmental contaminant exposures, citizen scientists were often recruited from communities disproportionately affected by environmental contaminants. These communities tended to be predominantly low income, with large racial/ethnic minority populations. Other stakeholders such as policy makers, teachers, business owners, and city officials were also included as citizen scientists in some cases and took part in activities that followed from the citizen scientists’ efforts (22, 56).

### Inclusion of Citizen Scientists in the Research Process

**Figure 1:**
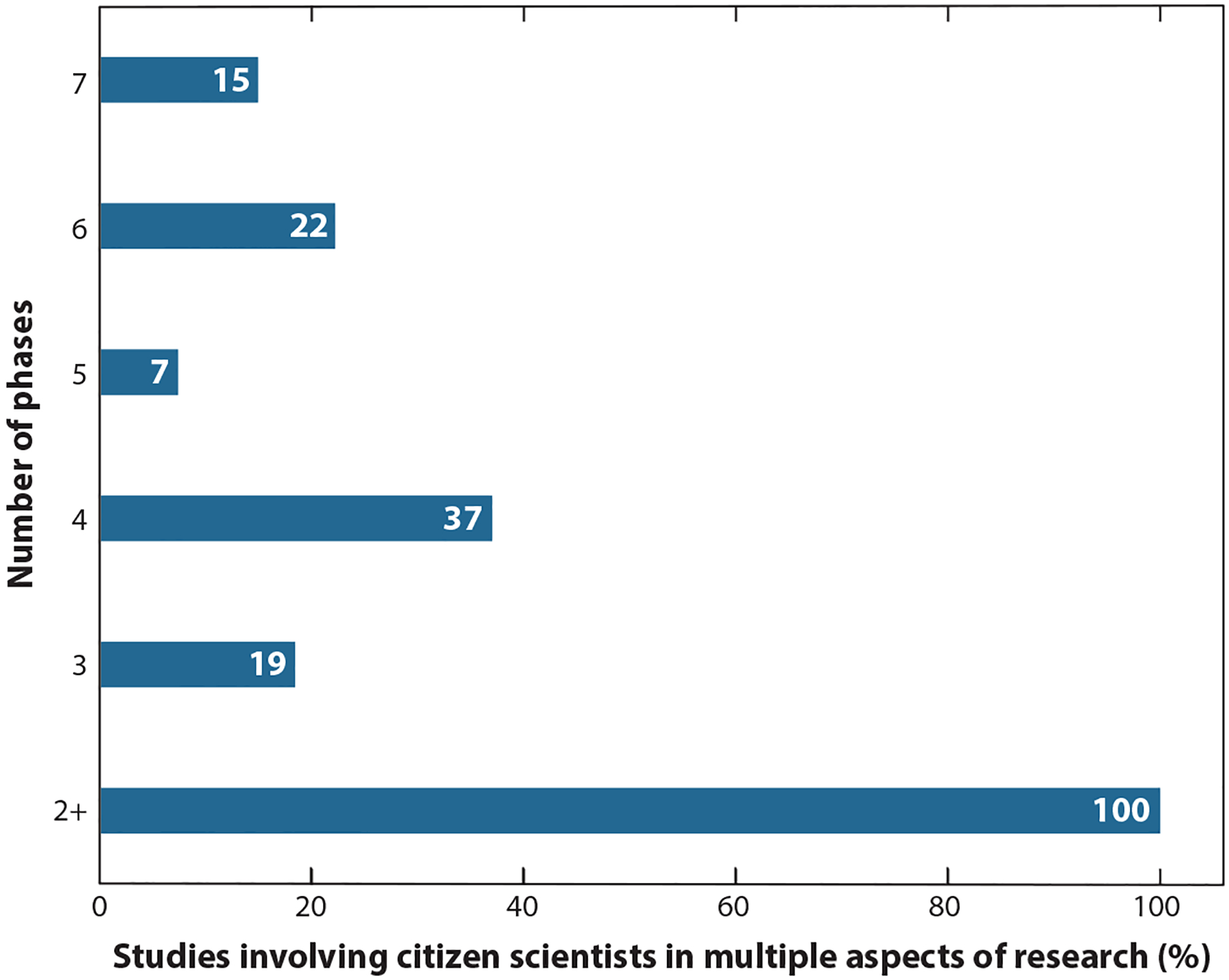
Percent of studies included in this review that involved citizen scientists in multiple aspects of research. Phases include defining the problem, defining the research question, designing the study, collecting data, analyzing data, interpreting findings, and disseminating/advocating for change.

**Figure 2:**
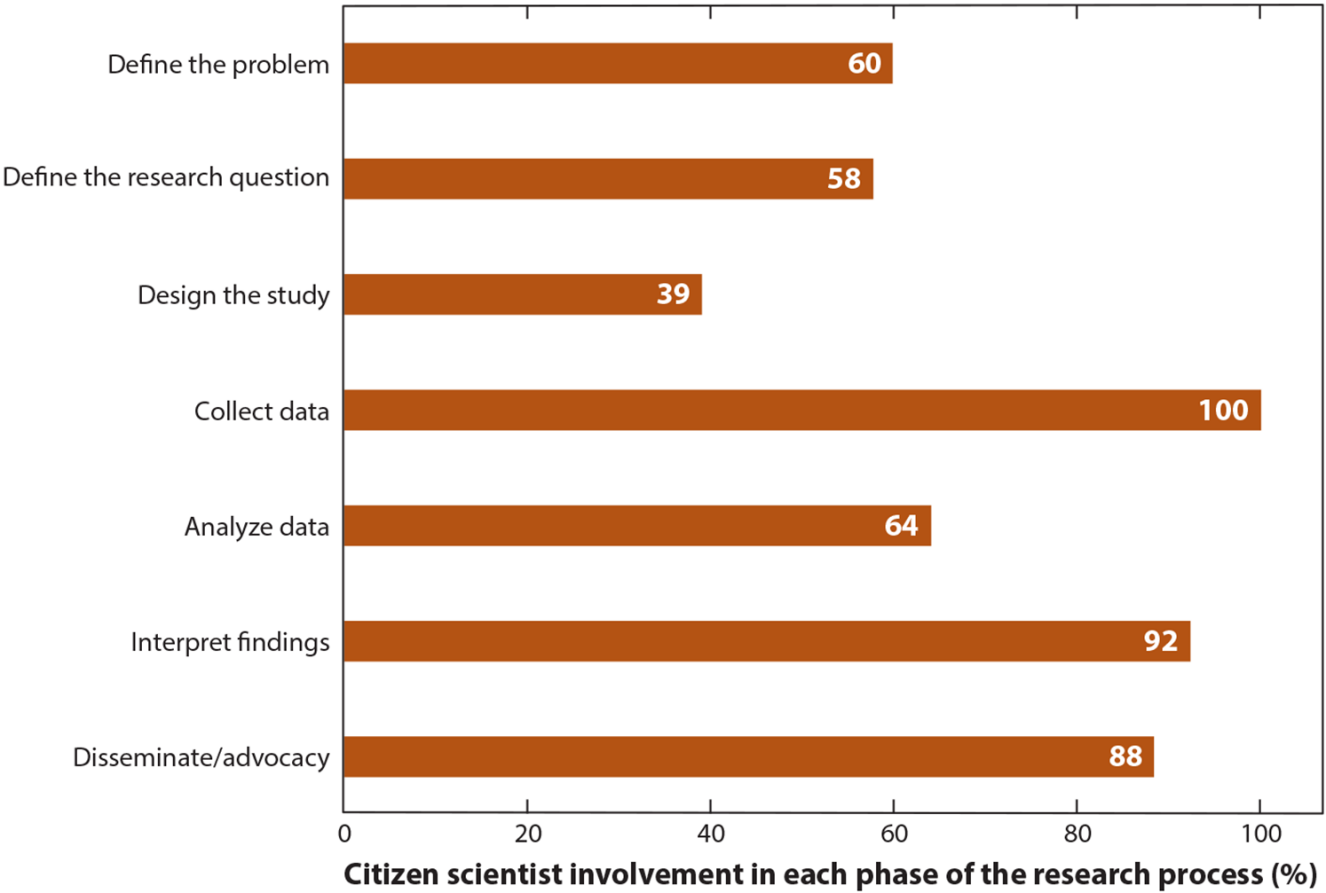
Percent of studies included in this review that involved citizen scientists in each phase of the research process.

As shown in Figure 1, 81% of studies included citizen scientists in four or more phases of the research process. (As noted above, studies that included citizen scientists only in data collection were excluded from this review.) These studies were most highly aligned with co-created, extreme, or “by the people” citizen science, as they involved citizen scientists throughout the research process. A smaller proportion of studies (19%) engaged citizen scientists in three or fewer phases of the research process. Citizen scientists were most commonly included in the data collection phase (Figure 2), a hallmark of citizen science. The next most common phases that citizen scientists were involved in were interpretation of findings and the dissemination/advocacy phase. Most studies involved citizen scientists in data interpretation and leveraged citizen scientists’ lived experience and local knowledge in data interpretation activities. Several studies described the process of providing individual research results (individual or community level) to citizen scientists and often included additional data to contextualize the results. For example, Jiao et al. (32) provided each resident with the level of arsenic in the soil around their home as well as average levels for the state and county. Several of the studies also included citizen scientists in activities where the data they collected were used to advocate for policy or program changes to improve health. For instance, Folkerth et al. (22) described how citizen scientists partnered with local leaders to use data they collected on indoor air quality to advocate for city-wide smoke-free policies.

Studies that included citizen scientists in the problem definition phase or in selecting the research topic (60% or 58%, respectively) were often part of a CBPR process where community partners and organizations had taken an active role. Few studies included citizen scientists in designing either the research study or the research tools. Similarly, citizen scientists were not typically involved in data analysis. For some studies, data analysis was conducted by researchers with advanced training in particular analytic techniques, such as laboratory assays for environmental contaminant exposures. However, other studies found ways to include citizen scientists in the data analysis process. Kim et al. (33) described how Native American youth took an active role in the analysis process by selecting key variables and deciding which associations to assess. In the *Our Voice* methodology, citizen scientists analyze their own data by viewing Discovery Tool–generated geotagged photographs and audio/text narratives as a group to identify themes and key issues to address.

### Evaluation of Citizen Science Approaches

We found substantial variability in evaluation of the citizen science process, particularly in the reporting of short-, mid-, and long-term outcomes or impacts (Table 1). While all the studies collected data relevant to the research area (e.g., data on environmental exposure to contaminants, built-environment barriers, or facilitators for physical activity or healthy nutrition) and reported on those data or on dissemination efforts, few indicated comprehensive or long-term evaluation of the project outcomes or of the processes and practices of engagement. Examples of evaluation efforts reported included prepost surveys assessing impacts on the citizen scientists themselves (26, 56). For example, Brickle & Evans-Agnew (4) assessed changes in empowerment, optimism, and interest in pursuing science education among youth as a result of participating in the study as citizen scientists. Prepost surveys were also employed by Zieff and colleagues (58) to assess changes in the built environment as a result of the project. Other evaluation efforts included qualitative data collection, such as interviews at various time points, to assess partnership processes and the effectiveness of the efforts in leading to sustained change (50, 57). Rodriguez and colleagues (44) employed a multimethod approach to measure impacts on student active travel (i.e., biking, walking) to elementary school by utilizing standard surveys, direct observation audits, and engagement measures collected at the start and end of the academic year. This study also involved a comparison school that did not receive the “by the people” citizen science program. This study demonstrated a between-arm difference in the rates of walking/biking to school at the end of the school year that was twice as high in the school receiving the additional citizen science program compared with the school receiving only the standard Safe Routes to School program (44).

## DISCUSSION

Significant advances in citizen science have enabled the inclusion of diverse community groups in the research process to address key aspects of the physical and social environments to promote health equity. Citizen science to promote health equity has used participatory science frameworks such as *Our Voice* and CHESS. As discussed above, citizen science approaches have been used to address exposures to environmental contaminants, physical activity, healthy eating, and other issues important for health equity. The use of mobile applications has enabled citizen scientists to collect robust data on their physical and social environments that they can then use to make important locally relevant changes. A diverse array of community members have been engaged as citizen scientists and involved in all phases of the research process. We provide the following recommendations to advance the field of citizen science to address health equity.

### Expand citizen science to focus on topics important for health equity.

There is great potential to involve citizen scientists in topics critical for promoting health equity. For example, systemic racism, health care access, transportation access, and housing stability are important determinants of health inequities that may benefit from the integration of citizen scientists. Engaging community members in addressing these important areas holds promise for advancing health equity through community-driven research and solutions.

### Increase the diversity of community members as citizen scientists, particularly those experiencing health inequities.

It is important to increase the diversity among people serving as citizen scientists to address topics critical to health equity. Future efforts should test citizen science recruitment methods, including random sampling and other approaches as appropriate, to better determine their impacts on the relevant environmental and community changes that are the primary goal of this research. Additionally, a focus on training citizen scientists as well as evaluating their experiences will offer opportunities to continually improve the experience for diverse citizen scientists.

### Increase the integration of citizen scientists in additional phases of research.

Our findings demonstrate that there are opportunities to integrate citizen scientists in additional phases of the research process, such as defining the research topic, designing the study, analyzing the data, and visualizing the data, to facilitate resident and stakeholder understanding and positive change. Including the perspectives of citizen scientists in all phases of the research process will likely confer additional advantages for promoting health equity.

### Continue to take advantage of emerging technologies that enable citizen scientists to collect, interpret, and present their data in compelling ways to advance health equity.

The field of citizen science has taken advantage of various technologies to enable community members to gather data in systematic and rigorous ways. Given the rapid evolution of technologies, the field will benefit from continued efforts to incorporate emerging approaches. For example, natural language processing (NLP) can be used to find patterns in participants’ comments and narratives to gather insights related to their sentiments, semantics, and frequency of use of certain language patterns and terms (8). This can be useful in synthesizing findings and evaluating the resident engagement process. Also, the use of NLP is helpful for scaling citizen science projects to larger groups and increasing the amount of information collected, as it amplifies the ability to gather insights from larger data sets. Another example is the use of natural language generation (NLG), which has been useful for motivating, training, and retaining community members involved in certain forms of citizen science (e.g., “with the people” citizen science, which has been used extensively in the natural and ecological sciences) by providing feedback on how correctly they implement research protocols. For example, in a study where citizen scientists volunteered to identify biological species using a photo-based citizen science program, the NLG feedback to participants consisted of providing the correct biological species to the volunteers along with the reasons the species was misidentified. This feedback in turn helped highlight the key features that facilitate correct identification (52). This process showed that the automated generation of informative feedback about identification accuracy fostered learning and engagement among the citizen scientists, increasing their productivity and retention for these types of “with the people” approaches. In contrast, for the “by the people” citizen science perspective emphasized in this review, where the data being collected directly reflect the perceptions of the community members themselves, there are no correct responses per se; rather, it is critical that the focus of the research remain primarily on the perspectives and insights of the community residents and stakeholders themselves.

Other accelerating technology areas that may find use in the participatory citizen science field include those that can help both residents and decision makers better imagine local changes that could positively affect community health. Beyond the photos that are often collected by citizen scientists, more robust, three-dimensional forms of visualization can be obtained from big data platforms such as Google Street View or Google Earth. In addition, applications of augmented reality or portable virtual reality platforms may provide more dynamic, immersive, and ultimately compelling ways to visualize potential changes suggested by citizen scientists.

### Strengthen the rigor of methods to evaluate the impact on health equity.

As citizen science for health equity continues to grow, increased attention is needed to ensure rigor in the evaluation of outcomes. The European Citizen Science Association proposed 10 principles of citizen science that include a focus on evaluation of citizen science programs for their “scientific output, data quality, participant experience, and wider societal or policy impact” (21). Participatory approaches may be particularly well suited to the evaluation of participant experiences and wider societal or policy impacts. One such participatory method is Ripple Effects Mapping (19, 53), in which researchers visually map citizen scientists’ reports of outcomes from their participation and how they came about. The process allows citizen scientists to share how intended or anticipated outcomes came about as well as to identify unintended or unanticipated outcomes. With the visual mapping that results from this process, the large array of impacts typically emanating from such participatory research can easily be communicated to diverse audiences, including future funders or local decision makers, to advocate for change.

Social network analysis of participating citizen scientists can also be integrated into process and outcome evaluations by capturing the formation and growth of networks among citizen scientists and stakeholders. Such real-world social networks are powerful for monitoring and capturing the interconnections of social environments that can drive community change (5, 29). Through social network analysis, it is possible to measure network cohesion over time and changes in social capital after a community’s participation in a citizen science project. Such measurement is useful for understanding the community’s involvement and the sustainability of the emergent networks as part of the citizen science process (15, 29). Also, the use of structural metrics makes it possible to identify agents of change and leaders in the network who can continue to leverage the citizen science process and follow-up activities after the citizen science project is finished (15, 30). This type of analysis also can be expanded to mechanistic models that allow for an understanding of the underlying characteristics of individuals related to the formation and dissolution of ties among participants. This can be useful for understanding the resilience of the network throughout the citizen science process and the network’s ability to promote changes in the environment.

Experimental designs can also be employed to evaluate the effectiveness of citizen science to positively influence physical and social environments as well as health outcomes. Cluster randomized trials are ideal for evaluating the effectiveness of citizen science approaches, given that their goals usually include changes beyond the individual level. Such designs are particularly useful for testing multilevel approaches that can combine individual-level interventions with a citizen science approach. For example, the National Institutes of Health–funded Steps for Change Trial has randomized affordable senior housing sites in the San Francisco Bay Area to receive a group-based physical activity intervention with or without integration of the *Our Voice* citizen science method. Housing sites that receive the group-based physical activity intervention plus *Our Voice* have been learning how to identify and address local environmental impacts on physical activity beyond the individual-level factors that have typically been the focus of physical activity interventions in many locales (34). Additionally, hybrid randomized trial designs can be employed when the goal is to evaluate effectiveness as well as potential for future implementation and dissemination. Hybrid trials assess effectiveness, similar to a traditional randomized trial, and add methods to assess implementation and dissemination (12, 17). Assessments of implementation and dissemination often use mixed methods and employ implementation frameworks such as RE-AIM (Reach, Effectiveness, Adoption, Implementation, and Maintenance) (24) and CFIR (Consolidated Framework for Implementation Research) (18). Such methods are ideal for evaluating how citizen science approaches could be widely implemented across other settings and populations.

Limitations of this review include the possibility that some studies employing citizen science to address health equity were inadvertently excluded. It is possible, for example, that some studies engaged community members in this type of participatory research process without naming it citizen science or using one of the other search terms employed in this review. Although other terms were included in our keyword search, most studies included in the final sample tended to use “citizen science” somewhere in their text. Published reviews of CBPR, Youth Participatory Action Research, Photovoice, and others have summarized bodies of research that share many qualities of citizen science (9, 41, 42). This review, in contrast, comments specifically on research using the term citizen science to characterize the involvement of individual community members in the research process as a strategy for addressing health equity.

## CONCLUSIONS

Citizen science is a powerful approach to address key determinants of health inequities, especially those that stem from adverse physical and social environment determinants. The use of citizen science to promote health equity commonly focuses on environmental contaminant exposures, healthy eating, physical activity, and their relevant physical and social environmental factors. Recommendations for advancing the field of citizen science to address health equity include (*a*) expanding its focus to critical topics for health equity; (*b*) increasing diversity among people serving as citizen scientists; (*c*) continuing to take advantage of emerging technologies to facilitate data collection, processing, and visualization; and (*d*) increasing the rigor of evaluation methods. Ultimately, the promise of citizen science to advance health equity lies in the opportunity this research method offers for empowering people who experience health inequities to both document and change the factors adversely affecting their health and the health of their communities.

## Supplementary Material

Supplemental material

## Figures and Tables

**Table 1:** Information on articles included in this review

Study	Demographics of citizen scientists	Brief description	Framework	Tools for data collection	Short-/mid-term outcomes	Long-term outcomes
**Environmental contaminant exposures**						
Ramirez-Andreotta et al. (43)	Community members (*n* = 43) in neighborhoods adjacent to contaminated sites in Arizona	Environmental sampling (e.g., soil, water, vegetables) to assess risk exposure of gardening and vegetable consumption from residential home gardens	CBPR	Tool kit for sample collection in home gardens	Exposure assessment (e.g., arsenic concentration) of residential soil samples; informal assessment of residents’ understanding of risk	Project served as initial step in community-level environmental efforts. Three new projects were initiated as a result.
Jiao et al. (32)	Residents (*n* = 42) of a diverse, low-income, underserved community in Ohio	Soil sampling and risk communication around industry environmental contaminants	Participatory Geographical Information Systems	PPGIS and Mappler X	Heavy-metal exposure assessments in residential soil samples and mapping tools of contributing factors	Plans to conduct future projects on air monitoring
Brickle & Evans-Agnew (4)	Diverse youth (*n* = 10) living in a variety of environments (urban, suburban, rural) in Washington State	Air sampling and advocacy for change in woodsmoke pollution; youth empowerment around environmental justice was also a central theme.	Youth empowerment	Questionnaires (e.g., around empowerment), air sampling, and picture documentation	Air samples were collected by youth in their homes. Community forum was attended by diverse community members.	Post survey assessments showed positive impacts on youth empowerment, optimism, and interest in becoming a scientist.
Sullivan et al. (50)	Gulf-fishing community members (*n* = 203) in coastal Louisiana, Mississippi, and Alabama	Environmental contaminant exposure measurements and community organizing after the *Deepwater Horizon* oil disaster	CBPR	Lab tools for collecting marine life samples	Qualitative interviews with hub coordinators at the project halfway point to assess efficacy and challenges of the citizen science process	A proposal was developed with community hub coordinators followed by community forums in the tristate region.
Haynes et al. (28)	Middle and high school teachers (*n* = 2) and community residents in the Appalachian region (number not stated)	Air and water quality sampling	CBPR	Water quality test kits	Air sampling, development of water test kit for schools, perceived usefulness of kits among teachers	Water sampling kits were incorporated into the schools’ environmental sciences curriculum. Efforts were covered by the local media.
Folkerth et al. (22)	Diverse stakeholders (*n* = 12) such as residents, advocates, and elected officials in a rural, low-income Kentucky community	Engagement in policy advocacy to reduce smoke/tobacco exposure	Not stated	AirBeam monitors that gather and graph air quality data	Assessed the impact of the project on tobacco policy; a successful smoke-free policy was passed by the city council.	Not stated
Hahn et al. (26)	Youth (*n* = 27) in rural and suburban communities	Radon testing and homeowner survey to promote awareness and education	Not stated	Radon testing tool kit	Post survey with youth about the capacity-building sessions to assess their knowledge	Not stated; youth are interested in future citizen science projects.
Newman et al. (39)	Residents, including high school students, of a marginalized neighborhood adjacent to industrial pollutants in Manchester, Texas (number not stated)	Environmental sampling (e.g., soil) to decrease environmental exposure to industrial pollution during floods	Not stated	Standard environmental sampling methods	Findings were used to develop a master plan to assist in reduction of exposure.	A model was developed to assess the performance of the master plan over the long term.
Wong et al. (57)	Primarily Latinx agricultural community residents (*n* = 45) on the US–Mexico border	Air monitoring project and establishment of a community air monitoring network, including data collection and trainings on community action planning	CBPR	Air quality monitors	Mixed methods were employed for evaluation, including key informant interviews at the conclusion of the project for process evaluation. Improved outcomes were reported across various domains (e.g., local knowledge, community networks, economic investment).	Formal evaluation reported elsewhere
**Physical activity and exercise**						
Rosas et al. (45)	Adults (*n* = 32) and youth (*n* = 9) in neighborhoods of diverse income and walkability in Mexico	Assessment of barriers and facilitators of active living to identify local and sustainable solutions	*Our Voice*	Discovery Tool plus interviewer-administered questionnaires (e.g., built-environment perceptions, self-efficacy, acceptability of the tool)	Showed the feasibility and acceptability of the data collection tool and citizen science approach in neighborhoods with a diverse range of incomes and limited tech literacy	Not stated
Winter et al. (55)	Latinx adolescents (*n* = 10) and older adults (*n* = 10) in a low-income urban community in California	Neighborhood environmental assessment around active living	*Our Voice*	Discovery Tool app plus interviewer-administered survey questions (e.g., around physical activity, neighborhood perception, social support)	Illustrated citizen scientists’ ability (with minimal training) to collect and analyze data and engage with stakeholders to improve their neighborhoods	Compiled a bilingual community resource guide for use by residents in addressing local issues; shared information on mitigating the effects of illegal dumping and trash from surrounding neighborhoods; worked with media sources to disseminate their activities
Tuckett et al. (51)	Adults 65 years of age or older (*n* = 8)	Assessment of physical features of the environment that hinder or promote physical activity; communication with decision makers for change	*Our Voice*	Discovery Tool app and brief questions around perceptions of neighborhood environment	Citizen scientists used findings to advocate for improvements: parks, footpath, and traffic safety.	Footpath repairs were begun; roadworks to fix road line markings to improve traffic safety were completed; and other construction projects, including construction of a new toilet block and additional exercise equipment in the local park, were approved.
Zieff et al. (58)	Multigenerational, multiethnic residents of three socioeconomically diverse cities in Colombia (*n* = 32), the USA (*n* = 10), and Chile (*n* = 8)	Assessment of Open Streets initiatives (e.g., temporary parks, street closures) to promote healthy living and physical activity in Latin America	*Our Voice*	Discovery Tool app plus survey instruments	Prepost surveys assessing built-environment features across two time points, with emphasis on testing acceptability and utility of the approach in the different settings	Insights into residents’ perceptions and behaviors surrounding urban Open Streets programs to set the stage for further citizen science research in Latin America
Rodriguez et al. (44)	Elementary school parents (*n* = 11) and middle school children (*n* = 26) in a primarily Latinx, lower-population-density community in California	Data collection and advocacy to strengthen Safe Routes to School (e.g., walking, biking) and increase active commuting	*Our Voice*	Discovery Tool app, surveys, and environmental observation audits	Engagement measures, environmental observations, and data on student travel mode were collected at the beginning and end of the school year to assess impact.	Following the first year, the school involved in the citizen science project had more engagement activities compared with the control site.
Odunitan-Wayas et al. (40)	Residents (*n* = 11) of a low-income urban South African community	Identification of barriers and promoters of physical activity that can become targets of advocacy efforts	*Our Voice*	Physical activity measures (self-reports, accelerometers), anthropometrie measures, and the Discovery Tool (with built-in collective efficacy questions)	Showcases feasibility of the approach in South Africa and citizen scientists’ ability to design locally relevant solutions	None stated. First-generation feasibility study to set the stage for further research in this area
Winter et al. (56)	Diverse stakeholders (e.g., residents, business owners) in the San Francisco Bay Area, California; citizen scientists (*n* = 9) included park users and local community residents.	Identification of various impacts (e.g., physical activity, tax revenue, socialization) of pop-up parks in urban areas, with recommendations	*Our Voice*	Direct observation, surveys, sales tax data, and key informant interviews with business owners and the Discovery Tool app	The Discovery Tool was used by citizen scientists on some locations to document before, during, and after perceptions and impacts of the local pop-up park, documenting primarily positive impacts.	Data collection in years 1,2, and 4 for longitudinal impacts
Rubio et al. (47)	Community leaders and community residents (*n* = 48) in two distinct neighborhoods in Bogotá, Colombia	Improving uptake and impact of a community-based publicly available physical activity program, Recreovía	*Our Voice*	Surveys, accelerometry data, anthropometric measures, and the Discovery Tool app	Efforts contributed to sustainability of the Recreovía program in an underresourced area that had been slated for elimination, and increased use of the Recreovía program. Another site was added.	Tracked long-term (6-month) outcomes including local capacity building and training of additional instructors
**Healthy eating and nutrition**						
Buman et al. (6)^a^	Older adults (*n* = 9–12 in two sites) who were residents of low-income communal housing sites in Northern California	Assessment and advocacy to improve environmental and policy around healthy eating and physical activity	*Our Voice*, participatory research	Discovery Tool, neighborhood audits (e.g., food outlet audit), and interviews with fellow low-income housing site residents	Tracking of community partnerships formed by citizen scientists as part of community-organizing activities	Initial impacts were started (e.g., garnering support for crosswalk, development of community garden). Longer-term outcomes are described by Winter et al. (54).
Akom et al. (1)	Underserved youth (*n* = 90) in Oakland, California	Youth mobilization for civic engagement, data collection, advocacy, and increased empowerment around food access and environmental justice	Youth Participatory Action Research	Streetwyze, a commercially available mobile app	Exit interviews with students following the study to understand impact on youth (e.g., self-esteem, academic engagement)	Additional projects were started as a result (e.g., student-run urban farm, establishment of food commissary).
Sheats et al. (48)	Racially/ethnically diverse older adults (*n* = 23) in a low-income urban community in California	Identification of solutions to barriers in accessing healthy food experienced by older adults; food environmental assessment	*Our Voice*	Discovery Tool	Tracked individual, short-term social-environmental effects (e.g., contacted decision makers about food issues)	Tracked long-term (up to 24 months post) built-environment improvements and advocacy efforts driven by citizen scientists in areas extending beyond food access, in what was termed ripple effects
Chrisinger et al. (14)	Adult residents (*n* = 8) of a high-poverty, high-unemployment city in New Jersey	Use of citizen science to evaluate impacts of a “healthy corner store” model on increasing access to healthy food and to positively influence health and well-being	*Our Voice*	Surveys (e.g., around perceived environment, food shopping preferences) and the Discovery Tool	Pilot study concentrated on feasibility of using the citizen science method in a low-income community.	Citizen scientists were invited to new community activities. The action steps developed and presented by citizen scientists to community leaders and stakeholders were incorporated into a planning document for a future Healthy Corner Store Network.
Hancock et al. (27)	Residents (*n* > 5,000) of small, low-income communities in England and Scotland	Assessment of barriers and assets around healthy eating to promote change and community-level interventions	CHESS	Mobile tool for Android tablets	Tracked individual-, environment-, society-, and policy-level changes	Additional efforts were undertaken to improve the CHESS tool for local communities.
Kim et al. (33)^a^	Native American youth (*n* = 12) in rural and remote Northern California	Community health and food assessment survey using a mobile app, primarily around nutrition, although included some physical activity work; youth also participated in leadership trainings and other activities.	CBPR	Community health and food assessment survey	Semistructured interviews at the end of the study	Several recommendations were implemented, including community gardens, improvements in food production, and securing additional funding.
**Other health promotion areas affected by social and physical environments**						
Garcia et al. (23)	African American and Latinx homeless youth (*n* = 15) in Los Angeles, California	Youth partnered with researchers and mentors to develop and administer a survey to youth regarding neighborhood problems, health, and educational issues.	CBPR	Survey administered to a convenience sample of youth identified as homeless	Tracking dissemination and advocacy efforts as well as policy changes, intended and unintended	Youth (now young adults) have continued to engage in advocacy and remain engaged with one another.
Chrisinger & King (13)	Adults (*n* = 14) in San Francisco Bay Area, California, recruited through a local nonprofit urban planning and design community partner	Identification of elements of the built environment that contribute to stress and well-being; suggestions of changes	*Our Voice*	Discovery Tool app and a wrist-worn biometric sensor, Empatica 4, that collected EDA	Found utility of adding a biometric sensor to standard *Our Voice* procedures for collecting neighborhood contextual information; significant associations were found between EDA levels and positively versus negatively rated photo clusters.	Not stated; the authors note the potential importance of combining both objective and perceived built-environment information from citizen scientists.
Chesser & Porter (10)	Adults (*n* = 10) age 65 or older who regularly visit or attend university (e.g., faculty, staff, community members) in Canada	Assessment and adoption of age-friendly environmental and social features in a university	*Our Voice*	Discovery Tool	Small pilot study documented issues and made recommendations for future institutional changes.	Additional efforts were later conducted following these methods and findings.
Chesser et al. (11)	Older adults (*n* = 10) who used university programs and facilities, such as retirees, faculty, staff, and students, in Canada	Identification of barriers and facilitators of age-friendly environments in a university, with recommendations for institutional change	*Our Voice*	Discovery Tool	Made recommendations in several areas, including for signage, accessibility, and transportation; documented the extent to which recommendations were included in current age-friendly university plans and priorities	Several findings (e.g., signage and accessibility concerns) are informing current campus wayfinding improvements.

a Studies dealing with multiple areas of research, particularly physical activity and healthy eating.

Abbreviations: CBPR, community-based participatory research; CHESS, Community Health Engagement Survey Solutions; Discovery Tool, Stanford Healthy Neighborhood Discovery Tool; EDA, electrodermal activity; PPGIS, Public Participatory Geographical Systems.
