# Supplementary material for: The Role of Citizen Science in Promoting Health Equity
Source: Annu Rev Public Health. Author manuscript; Available in PMC 2022 Apr 23. (PMC9034747; doi:10.1146/annurev-publhealth-090419-102856)
Supplement: Supplemental material [file NIHMS1798257-supplement-Supplemental_material.pdf]

Supplemental Material: Annu. Rev. Public Health 2022. 43.
https://doi.org/10.1146/annurev-publhealth-090419-102856
The Role of Citizen Science in Promoting Health Equity
Rosas, Rodriguez Espinosa, Montes Jimenez, and King

The Role of Citizen Science for Promoting Health Equity

Supplemental Materials

Supplemental Table 1. Technology tools employed in citizen science

Tools	Brief description	Additional information
Public Participatory Geographical Systems (PPGIS) and Mappler X	GIS used by citizen scientist, community-based organizations and others, often for social change and using publicly available mapping tools such as Mappler X.	https://www.participatorymethods.org/method/participatory-geographical-information-systems-pgis<br><br>https://sites.google.com/vertices.com/mapplernet/about/mappler-x-advanced-gis
AirBeam monitors	Wearable devices to gather, map, graph air quality data in real time. Requires minimal training of citizen scientists compared to other environmental exposure monitors.	https://www.habitatmap.org/aircasting
The Discovery Tool	Commercially available mobile app able employed by citizen scientists globally (with minimal training) to record geocoded pictures, audio and text narrative contextualizing the pictures. Tested with users 9-90+ in age and available in multiple languages.	https://med.stanford.edu/ourvoice.html
Streetwyze	Commercially available mobile application able to capture geocoded photos, audio, and video.	https://www.streetwyze.com
Community Health Engagement Survey Solutions (CHESS)	Includes a mobile mapping tool for Android tables. Intended for use by community residents and others in their neighborhoods. Includes a training component for the use of the tool.	https://www.c3health.org/our-programmes/communities/
